# Successful surgical treatment of omphalocele with umbilical evagination of the bladder: an extremely rare presentation of neonatal case

**DOI:** 10.1186/s40792-023-01710-y

**Published:** 2023-07-10

**Authors:** Haruka Kobayashi, Tokuro Baba, Masaaki Kuda, Satoshi Ieiri, Mitsuhisa Takatsuki

**Affiliations:** 1 Department of Digestive and General Surgery, Graduate School of Medicine, University of the Ryukyus, Nishihara, Japan; 2 Keiaikai Nakagami Hospital, Okinawa, Japan; 3 Department of Pediatric Surgery, Research Field in Medical and Health Sciences, Medical and Dental Area, Research and Education Assembly, Kagoshima University, 8-35-1, Sakuragaoka, Kagoshima City, 890-8520 Japan

**Keywords:** Omphalocele, Umbilical evagination of the bladder, Urachal aplasia

## Abstract

**Background:**

A few cases of small omphalocele with umbilical evagination of the bladder have been reported. However, its embryology is yet to be elucidated. Only a few reports have indicated the existence of urachal anomalies and umbilical cysts related to bladder evagination. The incidence of urachal anomalies at birth is reported to be 1 in 5000–8000 live birth, and urachal aplasia is rare. Herein, we report a rare, novel case of urachal aplasia.

**Case presentation:**

We encountered a small omphalocele with bladder evagination associated with urachal aplasia for which the neonate underwent surgery one day after birth. The patient was a one-day-old boy with a prenatally diagnosed omphalocele. A fetal magnetic resonance image (MRI) scan (25 weeks of gestation) revealed a 30 × 33 mm (approximately 1.3 in.) cystic lesion which was suspected to be an umbilical cyst. The baby was born vaginally at 38 weeks, weighing 2956 g. An omphalocele (hernial orifice diameter, 4 cm × 3 cm) with bladder prolapse was recognized. After sac excision, the prolapsed bladder was resected and closed with two-layer sutures. In order to secure sufficient bladder capacity, we estimated the minimum residual volume as 21 ml after bladder plasty. The remaining bladder capacity was confirmed to be 30 ml by injecting a contrast dye and saline into the bladder. The neonate had no associated cardiac urogenital or skeletal anomalies. Postoperative course was uneventful. The patient was regularly followed up for two years after surgery and underwent umbilicoplasty. He had no trouble with urinary function.

**Conclusion:**

In this case, we experienced extremely rare condition of a small omphalocele with bladder evagination associated with urachal aplasia and reviewed 7 case reports of anomalies similar to those in the present case. Umbilical cord cysts may be an informative indicator of these symptoms in utero. Therefore, ultrasonography scans should be conducted until delivery, despite the spontaneous disappearance of cord cysts.

## Background

The urachal anomalies occurrence at birth is reported to be 1 in 5000–8000 live birth, and urachal aplasia is extremely rare. Only a few reports have indicated the existence of urachal anomalies and umbilical cysts related to bladder evagination [1]. Herein, we report a rare, novel case of urachal aplasia in a neonate prenatally diagnosed with omphalocele and distinct fetal and neonatal characteristics.

## Case presentation

**Fig. 1:**
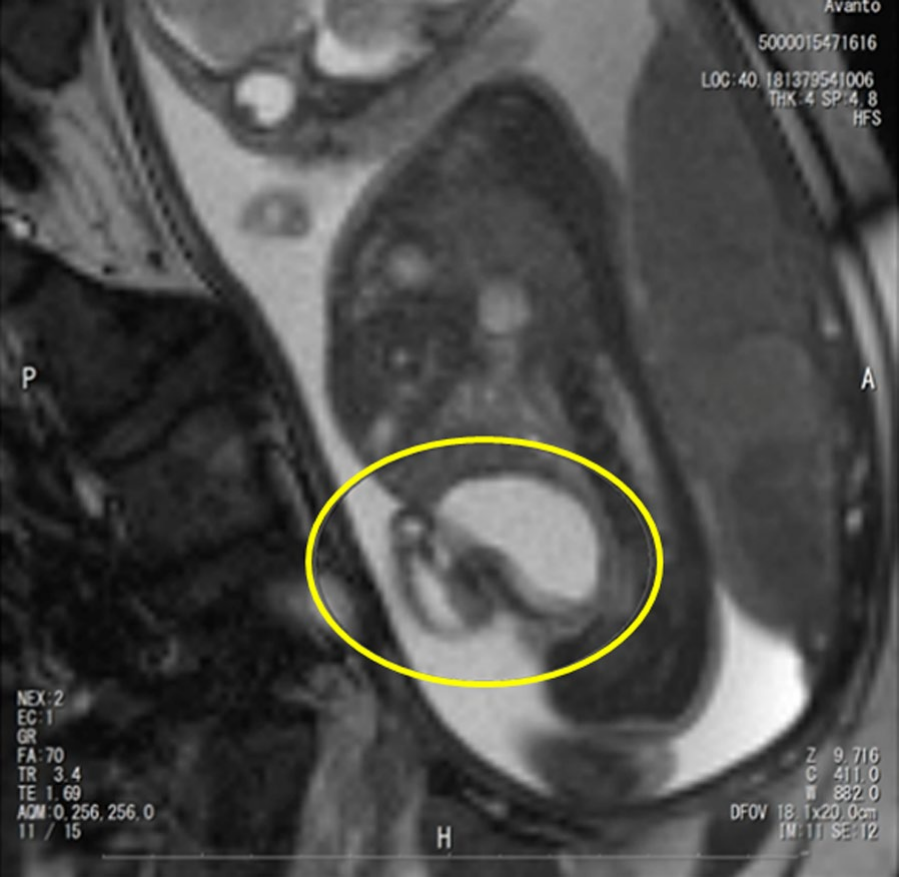
Fetal MRI finding of umbilical cyst at 25 weeks gestation. Yellow circle shows fetal bladder and umbilical cyst

**Fig. 2:**
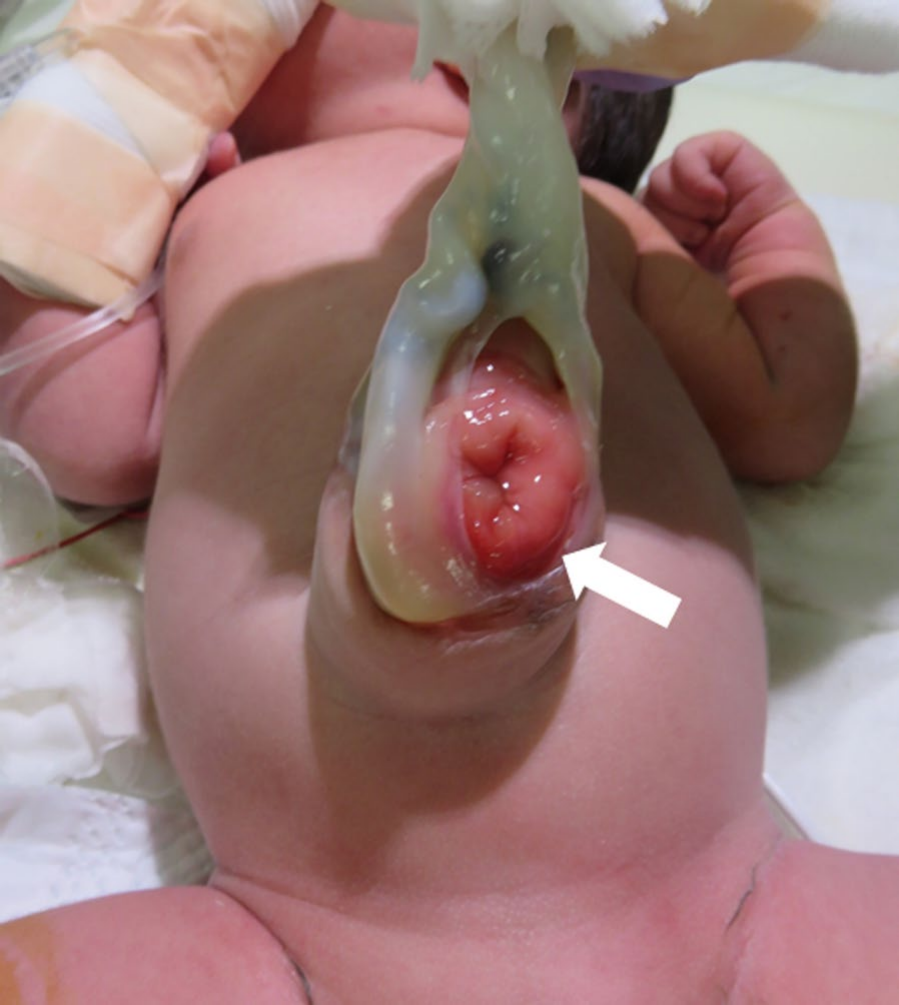
The appearance of bladder evagination and small omphalocele. White arrow shows bladder mucosa

The patient was a one-day-old boy with a prenatally diagnosed omphalocele. A 34-year-old pregnant mother was referred to our hospital at 17 weeks (approximately 4 months) of gestation with the chief concern of a singleton fetus with a 30 mm (approximately 1.18 in.) cystic lesion near the umbilical cord base. A fetal magnetic resonance image (MRI) scan (25 weeks of gestation) revealed a 30 × 33 mm (approximately 1.3 in.) cystic lesion as shown in Fig. 1 which was suspected to be an umbilical cyst. A follow-up MRI (at 34 weeks) revealed an umbilical cord hernia instead of a cystic lesion. The baby was born vaginally at 38 weeks, weighing 2956 g, with APGAR scores of 8 and 9 at 1 and 5 min, respectively. An omphalocele (hernial orifice diameter, 4 cm × 3 cm) with bladder prolapse was recognized as shown in Fig. 2. Based on appearance of umbilical site, physical examination and abdominal ultrasonography, the patient was diagnosed with a small omphalocele with bladder evagination. The small intestine prolapsed only while straining associated with crying, however, this was minimal, and we estimated that there would be low risk for hernia incarceration. So we planned the surgery for omphalocele with bladder evagination 1 day after birth.

### Operative procedure

**Fig. 3:**
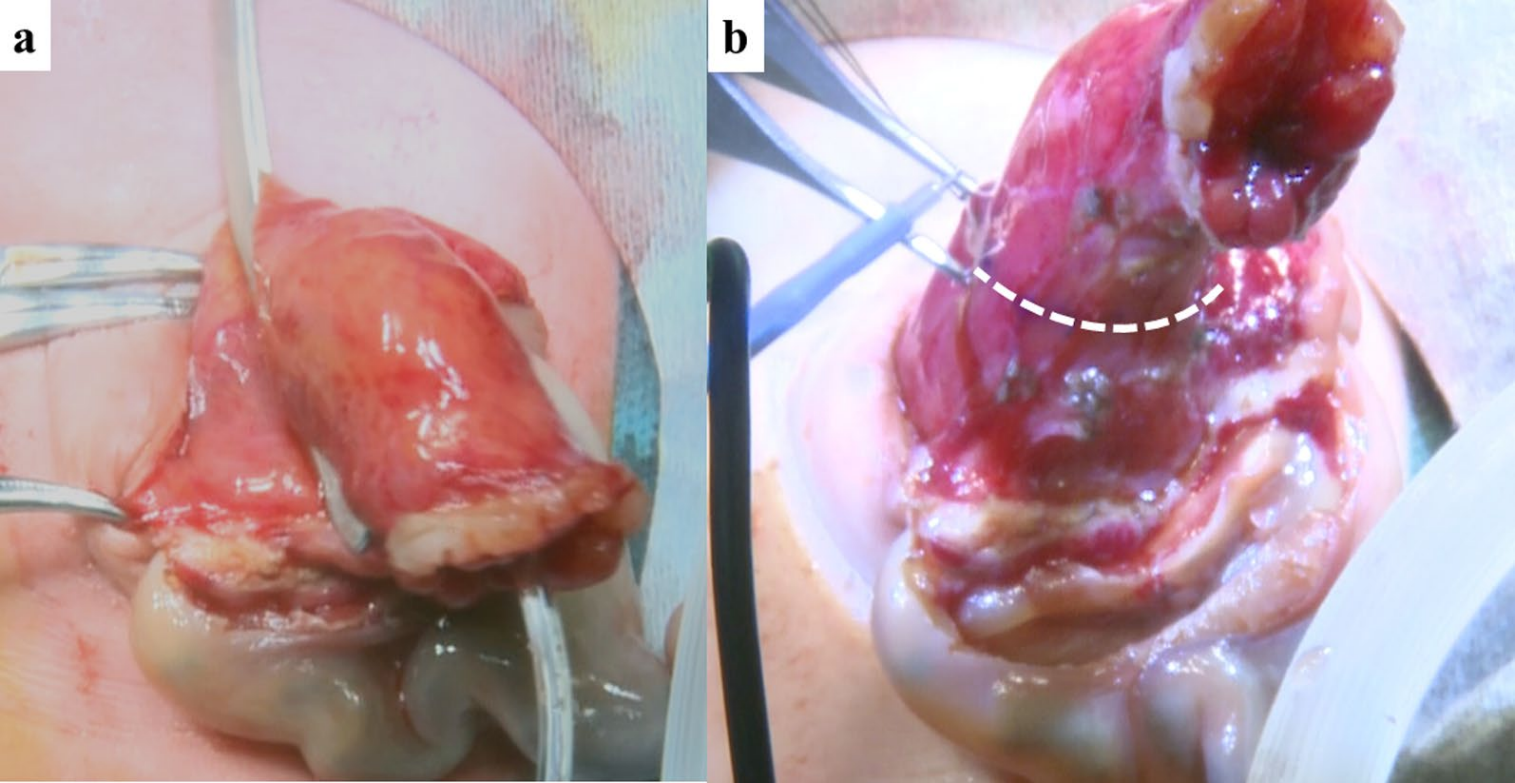
Measurement of residual bladder capacity and planned resection incision. **a** Residual bladder capacity determined to be 30ml via catherization, clamping, and instillation of contrast and saline. **b** White dotted line indicates the planned resection incision

Under general anesthesia, the patient was placed in supine position. After hernia sac excision, the intestine was naturally reducible and there were no other structural anomalies. In order to secure sufficient bladder volume, we estimated the minimum residual volume as 21 ml after bladder plasty. For bladder capacity, the simplified formula: Capacity (mL) = 7 × weight (kg) was a reliable estimate of expected bladder capacity in infants independent of age [2]. As shown in Fig. 3a, 8Fr. tube (Atom Multipurpose Tube, Atom Medical Corp, Tokyo, Japan) was inserted and the bladder was clamped as the maximum residual volume remained. And then clamped bladder was confirmed to be kept within the abdominal cavity. The bladder volume was also confirmed to be 30 ml using contrast medium. After the resection line was determined as shown in Fig. 3b, partial cystectomy was performed. The prolapsed bladder was resected and closed with two-layer continuous sutures with 4-0 PDS. The actual remaining bladder capacity was confirmed to be 30 ml by injecting a contrast dye and saline into the bladder. The patient had no associated cardiac, urogenital or skeletal anomalies. On pathological examination, bladder wall which was composed of mucosa and muscular layer was detected in the entire resected specimen.

### Postoperative course

**Fig. 4:**
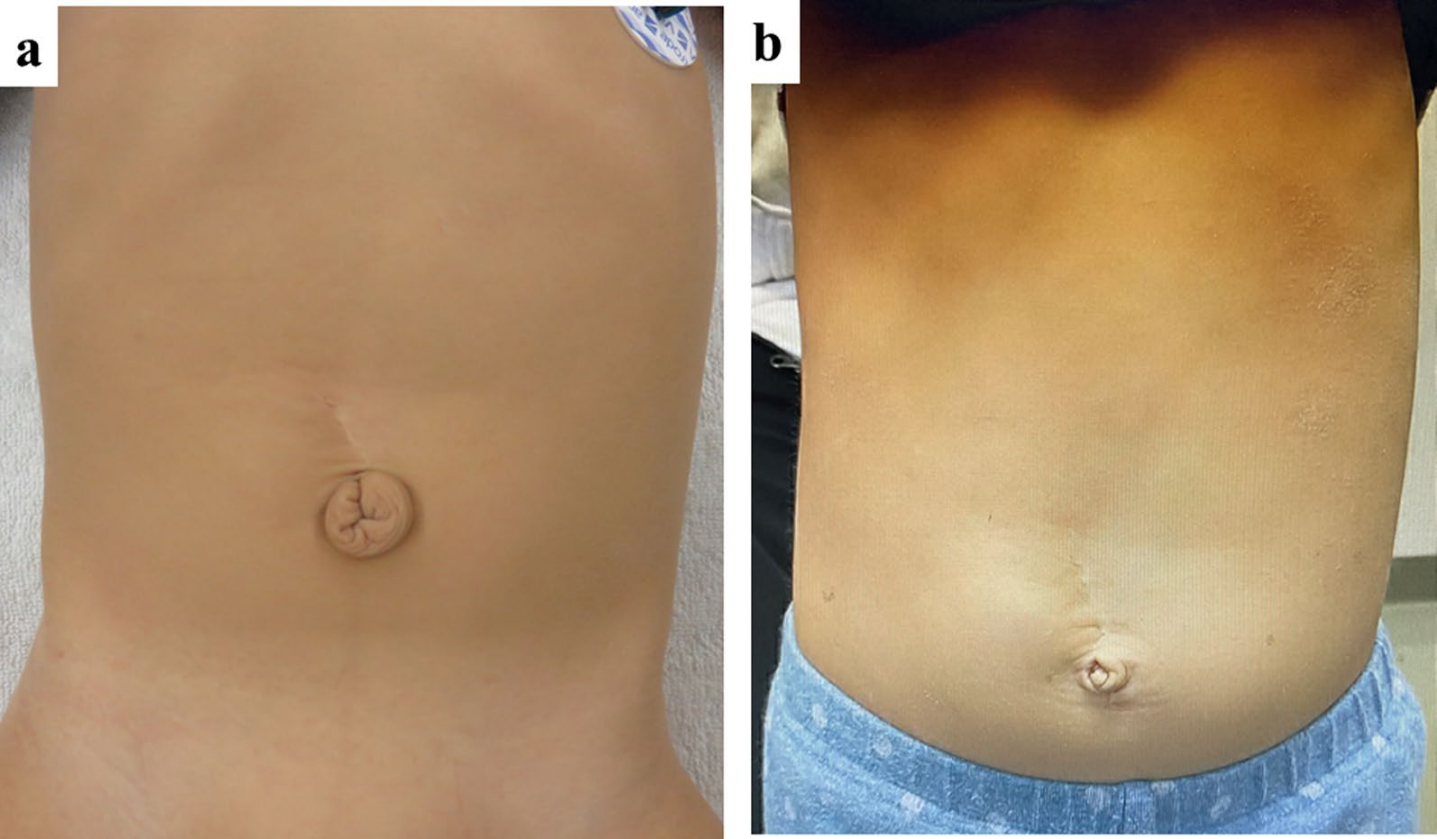
Umbilical appearance. **a** Umbilical appearance after initial surgery. **b** Umbilical appearance after umbilicoplasty

Postoperative course was uneventful. He was regularly followed up for 2 years after surgery (Fig. 4a), and underwent umbilicoplasty at 2 years old of age (Fig. 4b). He had no trouble with urinary function.

## Discussion

Here, we experienced and defined present case a small omphalocele with bladder evagination as a prolapsed bladder at the umbilicus with no significant abdominal wall defects and coexisting anomalies; the pelvic ring is intact and the genitalia are normal, which makes it distinct from bladder exstrophy. We reviewed seven cases of small omphaloceles with bladder evagination as shown in Table 1 [3–9]. In total, according to our research, only eight cases have been reported including our present case. Male patients (6: 75%) are dominant comparing with female patients (2: 25%). Three of the eight patients (37.5%) showed an umbilical cord cyst during the second or third trimester of pregnant period, which disappeared before delivery. Recently, prenatal diagnostic modalities such as ultrasonography and MRI have been developed, and umbilical cysts are easily detected [10]. Disappeared umbilical cord cysts in the second trimester may indicate bladder evagination with omphalocele. A follow-up ultrasonography is important, and it is essential to consult a pediatric surgeon to plan treatment strategy before delivery.

**Table 1:** Report of omphalocele with umbilical evagination of the bladder

Case no.	Author (year)	Sex	Prenatal diagnosis	Type of omphalocele	Associated anomalies	Age at surgery (Days)	Postoperative complications
1	Shimokawahara et al. (1978) [3]	Male	N.A	Cord hernia	Absent	36 days	Absent (after 20 months)
2	Takeuchi et al. (1981) [4]	Female	NA	Cord hernia	Absent	0 day	Absent (after 7 months)
3	Akashi et al. (1985) [5]	Male	NA	Cord hernia	Absent	0 day	Absent (after 1 month)
4	Grinda, et al. (1987) [6]	Male	NA	NA	Absent	0 day	A bilateral vesico-ureteral reflux grade II
5	Yaoita et al. (1994) [7]	Male	Umbilical cyst	Cord hernia	Absent	0 day	Perforative peritonitis
6	Thambi et al. (2000) [8]	Male	NA	Cord hernia	Absent	NA	NA
7	Werner et al. (2007) [9]	Female	Umbilical cyst / omphalocele	Cord hernia	Absent	2 days	Absent (after 5 years)
8	Our case	Male	Umbilical cyst / omphalocele	Cord hernia	Absent	1 day	Absent (after 25 month)

*NA* not applicable

The urachus is a structure lying between the peritoneum and the transversalis fascia, stretching from the anterior dome of the bladder to the umbilicus, located between the umbilical ligaments. A patent urachus is a complete connection between the bladder and umbilicus with a fully open ureteral duct which sometimes causes bladder prolapse. However, in the present case, not only the bladder but also the small intestine prolapsed from the umbilical cord. Furthermore, all the tissue of resected specimen submitted for pathological diagnosis revealed the bladder wall. Results of pathological examination indicated that the bladder did not prolapse through the urachus remnant, but through the umbilical cord [11]. From the view point of relationship between vesicoallantoic cyst and urachal aplasia, the probable etiology is that the umbilical ring was enlarged by vesicoallantoic cyst (gestation 25 week) and the cyst subsequently ruptured as urine output increasing (gestation 34 week) with development of the fetal abdominal wall, forming a cysto-umbilical fistula [7]. As a result, the bladder and small intestine prolapse into the expanded umbilical cord because of the increased intra-abdominal volume and pressure due to weight gain at birth. None of the patients had associated anomalies, indicating that evagination of the bladder was not associated with other syndromic disease.

Regarding the treatment after birth, umbilical cord hernia is the primary surgical indication, and surgical repair would be required in all cases. In our present case, the patient underwent surgical procedures of umbilical cord hernia repair, vesicourethral fistula resection, and bladder repair one day after birth. To determine the resection line of vesicourethral fistula resection, the sufficient bladder capacity should be left intact. However, the bladder should be resected because of the risk of carcinogenesis transformation from the residual ureteral duct tissue. Urachal masses are rare, but have the potential characteristics for malignant diseases. Thus, minimally invasive diagnostic and treatment strategy are strongly recommended to prevent the malignant transformation of large cysts in the early stages [12]. In our present case, the resection line was determined after confirming a bladder capacity of 30 ml using a contrast medium. For bladder capacity, the simplified formula: Capacity (mL) = 7 × weight (kg) was a reliable estimate of expected bladder capacity in infants independent of age [13]. In the present case, the capacity was: 7 × 2.956 kg = 20.692 ml, which was considered sufficient. Furthermore, the patient had no urological problems based on evaluation of ultrasonography, such as hydronephrosis, ureteral dilatation and bladder stone, for 3 years after surgery. In addition, the patient did not show urinary tract infection. But in the literature, a bilateral vesico-ureteral reflux grade II occurred in one case however, 6 months after, it is cured [4]. Perforative peritonitis occurred in one case [5]. No urinary problem was recognized in other 5 cases (Table 1).

Several authors suggest that systematic excision of urachal lesions detected during childhood should be performed to prevent infection or other problems in adulthood, such as the development of malignancy in residual remnants [12]. Of adult urachal anomalies, 51% were malignant and cancer risk increased with advancing adult age [13]. Because of potential malignancy, urachal anomalies should be removed when definitive diagnosis was obtained. Further research would be required to investigate future malignant change of residual remnants in infantile cases. To detect the urachus anomaly, ultrasonography was the most commonly used imaging modality, followed by fluoroscopy/VCUG, CT and MRI [14].

In the present case, the specimen did not show urachal histology. However, if an urachal anomaly is not resected in childhood, the pediatric surgeon and patient should keep in mind regarding the potential future risk of malignancy and the need for lifelong screening. If bladder capacity cannot be secured at the time of initial surgery, the augmentation cystoplasty using stomach and small intestine should be considered in the future. Augmentation cystoplasty is a surgical intervention that can significantly and continuously improve the quality of life in children. However, careful patient selection and appropriate preoperative counseling are essential, given the potential complications of urinary leakage and urinary tract stone disease and the fact that augmentation cystoplasty is an independent risk factor for bladder malignancy [15].

Regarding the design of the umbilical ring, a new ring was formed on the caudal side, with the umbilical artery and cord remaining in the center, which was expected to be retracted and recessed in the future [16]. Here, the umbilicus was not retracted; however, reconstruction of the umbilicus was performed using part of the umbilical cord.

## Conclusion

We experienced extremely rare case of a small omphalocele with umbilical evagination of the bladder caused by an umbilical cord cyst combined with urachal aplasia. Umbilical cord cysts may be an informative indicator of these symptoms before delivery. Therefore, ultrasonography scans should be conducted until delivery, despite the spontaneous disappearance of cord cysts. Furthermore, umbilical cord hernia is the primary surgical indication and we recommend to determine the resection line after confirming a bladder capacity of over 7 × weight (kg) in infants.

## Data Availability

The data supporting the findings of this study are available from the corresponding author upon reasonable request.
